# Meteorological Factors and the COVID-19 Pandemic: The Backdrop of Pakistan

**DOI:** 10.3389/fpsyg.2021.764016

**Published:** 2021-11-25

**Authors:** Muhammad Riaz, Muhammad Nadeem Akhtar, Shu Jinghong, Habib Gul

**Affiliations:** ^1^School of International Trade and Economics, University of International Business and Economics, Beijing, China; ^2^Department of Business Administration, Kardan University, Kabul, Afghanistan

**Keywords:** ARDL, COVID-19, weather, indicators, Pakistan

## Abstract

Coronavirus victims have been confirmed all around the world and millions of people are being put into self-isolation. In this backdrop, a superior appreciation of the effective parameters in epidemic spreading can cause a cogent assessment toward COVID-19. In this vein, the consequences of weather indicators on the spread of COVID-19 can play an instrumental role in the current coronavirus situation enveloping the world. These elements entail time, maximum and minimum temperature, humidity, wind speed, and rainfall. By such an incorporation, their consequent effects on coronavirus in Pakistan are explored. In the current study, principal elements are considered including the number of infected patients with coronavirus in Pakistan. The autoregressive distribution lag (ARDL) approach is used to analyze the effects and relationships of variables with the COVID-19 expansion rate extracting data from April 1, 2020 to April 30, 2021. The results revealed that maximum and minimum temperature, humidity, wind speed, and rainfall had a significant positive correlation with total and confirmed cases of COVID-19. Lastly, this brief communication attempts to clarify the outbreak of coronavirus in the region.

## Introduction

**FIGURE 1:**
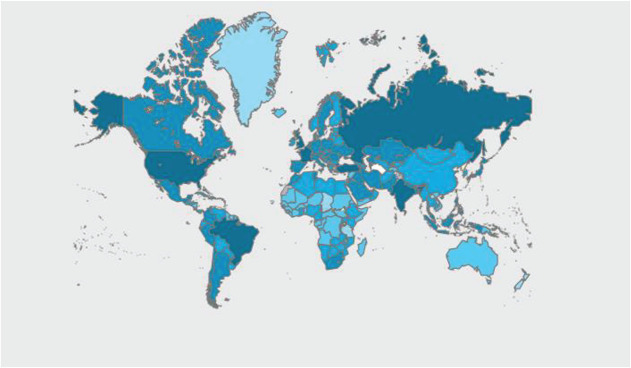
Total cases (WHO: April 30, 2021).

In December 2019, the World Health Organization (WHO) collected details about an endemic outbreak with anonymous detection (Deepak and Ameer, 2020) in Wuhan, Hubei province, China (Zhu et al., 2020). On February 2020, this epidemic was formally named COVID-19 predicating upon the detection of acute respiratory syndrome coronavirus-2 (SARS-CoV-2). Being a contagious disease, COVID-19 affected people all over the world. The main objective of this study is to investigate the number of total and confirmed cases in Pakistan by collecting information from April 1, 2020 to April 30, 2021. However, we also investigate the relationship between COVID variables (total cases and active cases) and environmental factors (temperature, humidity, and rainfall). WHO announced that total confirmed cases reached 187,284,207 and deaths reached 4,077,612 worldwide at the end of April 30, 2021 (Figure 1).

The study of Andersen et al. (2020) concerning COVID-19 revealed that most patients suffer from difficulty in breathing and pneumonia. Some scholars (Holshue et al., 2020; Perlman, 2020) reported this syndrome to be akin to other coronavirus illnesses like SARS and MERS, in which struggling to breathe due to respiratory distress was apparent. In an unpleasant case scenario, COVID-19 affected kidney disorder, caused pneumonia, and resulted in death as shown in the study of Wang et al. (2020). In Pakistan, two cases of COVID-19 were recorded in Karachi on February 26, 2020. Later, a myriad of cases were registered in all provinces and Islamabad capital territory on March 18, 2020, gradually encapsulating all districts in the country by June 2020. Consequently, Pakistan was proclaimed to have the second highest number of confirmed patients in South Asia (after India). On April 30, 2021, new daily active case numbers reached their peak, with 91,547 new cases reported. As the capital of Sindh, Karachi is one of the largest cities in Pakistan. It is situated along the coastline of Sindh province in southern Pakistan, along the Karachi harbor, a natural harbor on the Arabian Sea. In Karachi, the summers are hot, oppressive, arid, and windy; the winters are short, comfortable, and dry; and it is mostly clear year-round. Over the course of the year, the temperature typically varies from 55°F to 94°F and is rarely below 49°F or above 100°F. Karachi covers an area of 3,780 km^2^ and a population of over 16 million (as of 2020). Even though death may be influenced by numerous elements, the current study is to explore the results from meteorological variables on COVID-19 deaths.

In Pakistan, the number of COVID-19 active cases has risen rapidly. As a result, the government of Pakistan has halted transport and trade with Iran (i.e., the major exporter of COVID-19). The land borders with China and India have also been blocked. All trading activities are controlled at the international boundaries. The perils of virus transmission in Pakistan are very high and require acceptable precautions and robust steps to quickly identify possible cases and fast surveillance to prevent further virus transference. In this approach, the number of cases might not increase concurrently with the increases all over the world and so far, it is implausible to find the point of inception. With the increase in patients of extremely infectious COVID-19, the Pakistan economy is now under decline. The terror of calamitous diseases and economic distress have combined. The nation cannot tolerate extended lockdowns and should the lockdown extend, Pakistan has already tolerated unmanageable economic loss. Pakistan does not have any acceptable resources to provide for patients at this time. Most of the people work on daily wages. Complete lockdown of the country could cause death either due to hunger or COVID-19. In the wake of such economic limitations, it has been decided that, instead of complete lockdown, people should avoid group meetings, and partial lockdown of the country will take place in order for the economy to provide for essential workers.

## The Present Situation in Pakistan

The Islamic Republic of Pakistan, the fifth most populous country with a population exceeding 225.2 million, is a country situated in South Asia. Pakistan is the 33rd largest country by area, spanning 881,913 square kilometers (340,509 square miles). As a federation, it has five provinces, namely, Punjab, Sindh, Balochistan, Khyber Pakhtunkhwa (KPK), and Gilgit-Baltistan (GB), and Islamabad as a capital city. As reported by the Ministry of Health (MoH), Government of Pakistan (GoP), there have been 820,823 confirmed cases, 91,547 active cases, 17,811 deaths, and 711,465 recoveries up until April 30, 2021 in the country. Most of the cases are reported in the Punjab province tolling 301,114 cases followed by Sindh (282,445), KPK (117,557), Islamabad (75,067), Balochistan (22,278), AJK (17,057), and GB (5,305). The outcomes of these cases are demonstrated in Table 1. According to this, the number of deaths recorded in Punjab province is 8,410, followed by Sindh (4,633), KPK (3,274), Islamabad (679), AJK (475), Balochistan (234), and GB (106). Similarly, the total number of recovered infected people in Sindh province is 264,052, followed by Punjab (243,463), KPK (102,142), Islamabad (61,786), Balochistan (20,592), AJK (14,359), and GB (5,072). The death rate in Pakistan remained 2.2% with a recovery rate of 86.7% on April 30, 2021.

**TABLE 1:** COVID-19 cases in Pakistan (April 30, 2021).

**Provinces**	**Total cases**	**Confirmed cases**	**Deaths**	**Recoveries**
AJK	17,050	2,223	475	14,359
Balochistan	22,278	1,452	234	20,592
GB	5,305	127	106	5,072
Islamabad	75,067	12,603	679	61,785
KPK	117,557	12,141	3,274	102,142
Punjab	301,114	49,241	8,410	243,463
Sindh	282,445	13,760	4,633	265,052

*Ministry of Health, Government of Pakistan.*

## Literature Review

Recently, a few studies have contemplated COVID-19 vis-à-vis environmental factors. The study of Ahmadi et al. (2020) explains metrological variables (e.g., humidity, wind speed, and average temperature) in climate particles in Iran based upon evidence from February 19 to March 22, 2020. Similarly, the seminal work of Andersen et al. (2020) covers SARS-CoV-2, which is deemed the seventh coronavirus known so far and can cause severe diseases. Furthermore, the work of Asyary and Veruswati (2020) reports the total deaths and total recoveries in Indonesia from March 2 to April 10, 2020. The analysis of Bashir et al. (2020), regarding a UK dataset collected from March 1, 2020 to April 12, 2020 from the health sector, described effects of environmental parameters such as minimum, maximum, and average temperature, humidity, wind speed, and rainfall in New York City. In extension, the analysis of Ma et al. (2020) recounts how meteorological parameters are important factors influencing infectious diseases (e.g., high fever, cough, and SARS). The model (Ma et al., 2020) explains the impact of temperature, humidity, and diurnal temperature range on a daily basis. Additionally, the work of Abid et al. (2020) describes the COVID-19 epidemic in Pakistan from the first day (i.e., February 25, 2020 to April 10, 2020) taking 45 days of epidemic data containing 4,601 confirmed cases, 46 deaths, and 727 recovered patients.

The review of Meo et al. (2020) defines humidity and temperature based on recordings from December 29 to May 12, 2020 in the top 10 hottest and coldest countries in the world. Similarly, the survey of Gorbalenya et al. (2020) explains classifying 2019-nCoV and naming it SARS-CoV-2, along with the work of Gupta et al. (2020), which discloses the significance of geographical factors of the COVID-19 outbreak in India. The study applied long-term climate records of air temperature, rainfall, humidity, and population density at the regional level for inspection. Furthermore, analysis of Qi et al. (2020) describes humidity and temperature in China with the help of time series analysis in Hubei from December 1, 2019 to February 11, 2020, and in other provinces from January 20 to February 11, 2020, as well as the review of Singhal (2020) which inspects the Review of Coronavirus Disease-19 in Wuhan and Hubei provinces. The survey of Tosepu et al. (2020) recounts the relationship between the environment and COVID-19 in Jakarta, Indonesia for the time period January 1, 2020 to March 29, 2020, data were obtained from the official website of Ministry of Health of the Republic of Indonesia Metrological department. Data consisted of minimum, maximum, and average temperature, humidity, and rainfall.

## Data Description and Methodology

### Data Collection

Data from April 1, 2020 to April 30, 2021 in Pakistan were collected including weekly total active, recovered, and death cases of COVID-19 grounding on meteorological data. Weekly death numbers of COVID-19 were collected from the official website of the Ministry of National Health Services, Government of Pakistan. The meteorological data were obtained from the National Weather Forecasting Center Islamabad and Pakistan Meteorological Department Government of Pakistan. Climate indicators included weekly temperature, wind speed, humidity, and rainfall. There are a number of studies in this context; therefore, we followed recent literature and calls (Ahmadi et al., 2020; Bashir et al., 2020; Meo et al., 2020) to develop the current model.

### Methodology


(1) CC=β+β⁢1⁢t⁢m⁢a⁢x+β⁢2⁢t⁢m⁢i⁢n+β⁢3⁢w⁢s+β⁢4⁢h⁢d+β⁢5⁢rf+ϵ

(2) AC=β⁢0+β⁢1⁢t⁢m⁢a⁢x+β⁢2⁢t⁢m⁢i⁢n+β⁢3⁢w⁢s+β⁢4⁢h⁢d+β⁢5⁢rf+ϵ

Where CC represents confirmed cases, AC means active cases, tmax equals maximum temperature, tmin stands for minimum temperature, ws represents wind speed, hd is humidity, rf means rainfall, β_*0*_ is the constant, β_*1,2,3,4,5*_ are coefficients, and ϵ is the error term.

To choose the design for time series evidence, stationarity data are inexorable. Stationarity data are first reported by utilizing peer-group unit root tests, augmented Dickey–Fuller (ADF), and Phillips Pearson (PP). The ARDL bound test is utilized to calculate co-integration among total active cases, maximum and minimum temperature, wind speed, and rainfall. If the variables are integrated at 1(0) then ARDL is the most acceptable technique. An early step regarding ARDL co-integration is the choice of lag length. In the current study, Akaike information criterion (AIC) is utilized, as a number of other scholars also utilized this approach such as Manzoor et al. (2019) and Danish and Wang (2019). Furthermore, the ARDL model examined the co-integration given among variables. In the robustness test, we used CUSUM and CUSUM of Squares for model validation.

## Results and Discussion

Table 2 describes the outcomes of the unit root tests. The given evidence reveals that all information in Table 2 is stationary at 1(0) and 1(1). The bound test is utilized to convince aspirations that the *F*-values are higher in the upper bound which is confirmation of co-integration. The outcome can be seen in Table 3. After the confirmation of the bound test of the co-integration in the modeled variables, the next step is to calculate the short-run (Table 4) and long-run (Table 5) co-integration by combining the real minimum and maximum temperature, wind speed, humidity, and rainfall with the total and confirmed cases.

**TABLE 2:** Unit root test.

**Variables**	**ADF**		**PP**	
	**At level difference**	**First**	**At level difference**	**First**
AC	−3.226 (0.023)** 2.644 (0.090)	−	−2.083 (0.014)*** 2.621 (0.095)	−
CC	−2.153 (0.015)*** 1.421 (0.056)	−	−9.123 (0.000)*** 2.360 (0.069)	−
Tmax	−2.006 (0.283) 8.745 (0.000)***	−	−1.883 (0.337) 8.315 (0.000)***	−
Tmin	−1.013 (0.742) 8.427 (0.000)***	−	−1.203 (0.667) 8.222 (0.000)***	−
Ws	−6.493 (0.000)*** 1.027 (0.451)	−	−6.566 (0.000)*** 2.219 (0.065)	−
Hd	−8.745 (0.000)*** 2.670 (0.085)	−	−2.670 (0.085) 9.391 (0.000)***	−
Rf	−3.112 (0.031) 9.113 (0.000)***	–	−3.039 (0.037) 9.862 (0.000)***	−

Parentheses () show *p*-value results, significant at 1% ***, at 5% **, and at 10% *. ADF, augmented Dickey–Fuller; PP, Phillips Pearson.

**TABLE 3:** ARDL bound test.

**Equation**	***F*-statistic**	**Decision**
AC = *f*(β0 + β1*tmax* + β2*tmin* + β3*ws* + β4*hd* + β5*rf* + ϵ)	5.7504	Integration
CC = *f*(β0 + β1*tmax* + β2*tmin* + β3*ws* + β4*hd* + β5*rf* + ϵ)	5.9216	

*ARDL co-integration.*

**TABLE 4:** Short-run coefficients.

**Variables**	**Model 1**		**Model 2**	
	**Coefficients**	***P*-values**	**Coefficients**	***P*-values**
Tmax	−0.433	0.017***	−0.087	0.035**
Tmin	0.330	0.040**	0.071	0.029**
Ws	−2.223	0.035**	−0.025	0.030**
Hd	−0.1423	0.017***	−0.026	0.021**
Rf	−0.169	0.012***	−0.029	0.002***

*P*-value results, significant at 1% ***, at 5% **, and at 10% *.

**TABLE 5:** Long-run coefficients.

**Variables**	**Model 1**		**Model 2**	
	**Coefficients**	***P*-values**	**Coefficients**	***P*-values**
Tmax	−3.342	0.014***	−0.825	0.013***
Tmin	2.938	0.035**	0.834	0.046**
Ws	−1.585	0.045**	−0.886	0.035**
Hd	−0.929	0.017***	−2.275	0.016***
Rf	−2.795	0.000***	−1.322	0.000***

*P*-value results, significant at 1% ***, at 5% **, and at 10% *.

COVID-19 has created health issues all over the world (Ma et al., 2020). In the present work, we described the spatial correlation with long-term and short-term climate and environmental factors with the counts of active cases of COVID-19 in Pakistan. The study of Meo et al. (2020) and Sajadi (2020) used different places around the world to examine the relationship between COVID-19 and weather situations. According to Tosepu et al. (2020), inducing aerology (humidity, wind speed, temperature, and rainfall) naturally adjusted environmental stability, therefore it might be affecting the sustainability of viruses.

The current study reported weekly new cases and confirmed total and active cases in Pakistan from April 1, 2020, onward. The first week started from April 1, 2020 to April 7, 2020. The active cases numbered 3,549 in the first week which rose to 4,432 the week ending April 14, 2020. And at the close of April 30, 2021, the active cases had increased to 91,547. In the present study, the maximum and minimum temperatures were 37 F^0^ and 13 F^0^, respectively. The highest humidity was 89% (lowest humidity, 35%), the lowest wind speed was 7 mph (highest wind speed, 33 mph), and the lowest rainfall was 0 mm (highest rainfall, 0.6 mm). Maximum and minimum temperatures were significant for total and active cases; wind speed, humidity, and rainfall were also significant for active cases and total cases in Pakistan. Our conclusions showed that maximum and minimum temperature, wind speed, humidity, and rainfall were positively related with dependent variables. Past work of Tan et al. (2005) and Vandini et al. (2013) supported our conclusions. Furthermore, the study of Shi et al. (2020) described weather parameters and declared that atmospheric pressure was a driver for the coronavirus disease.

Temperature along with humidity also played a key role in the occasional expansion of SARS-CoV-2 as Sajadi (2020) also announced the same conclusions for cases in China. Similarly, the study of Ma et al. (2020) advised that a moist climate also played a key role in the mortality rate from COVID-19, as environmental parameters and temperature were associated with the expansion of COVID-19.

### Robustness Analysis

**FIGURE 2:**
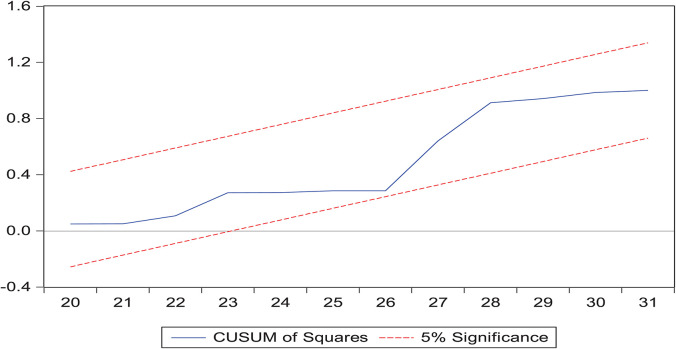
Cumulative sum of residuals.

**FIGURE 3:**
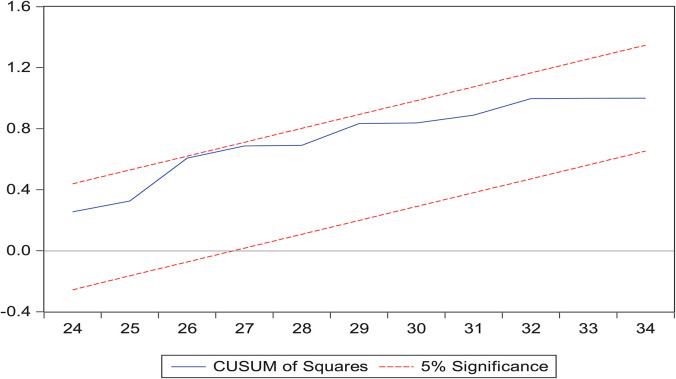
Cumulative sum of squares of residuals.

This work explains the “cumulative sum of residuals (CUSUM)” and the “cumulative sum of the square of residuals (CUSUM2)” through analysis of the fitness of the model. Figures 2, 3 demonstrate the results through a graphical representation of the model fitness. The red bar displays the border of the key area and the blue line is the indicator of the key area. If the blue line is inside between both red lines, this means the model is confirmed and stable. After confirmation, co-integration was completed among the total cases and other climate indicators.

## Conclusion

Environmental factors are playing a key role in the fight facing COVID-19 in Pakistan. The autoregressive distribution lag (ARDL) approach was used to identify the outcomes in the context of Pakistan. Findings indicate that maximum and minimum temperature, humidity, wind speed, and rainfall had a positive and significant association with total and confirmed cases affected by COVID in Pakistan from April 1, 2020 to April 30, 2021. Scientists are working to originate treatments and vaccines to avert this epidemic. Concurrently, if we start to initiate quarantine, it could rescue society as a whole and the risk will reduce directly. This is a condition where everyone has to cooperate to keep the risk down by staying at home. Although the current study provides solid proof of a correlation between weather indicators and COVID-19, the following constraints should be noted. Initially, further analysis is required. As SARS-CoV-2 is a contagious infection, further variables, such as social distancing, people’s tolerance, and the opportunity for well-being facilities, should be considered. Finally, awareness about personal hygienic measurements like wearing a mask and hand washing need to be probed further in future investigations.

## Data Availability Statement

The datasets presented in this article are not readily available because according to Country law and Kardan University data Sharing Policy. Requests to access the datasets should be directed to HG, habibgul544@yahoo.com.

## Ethics Statement

The studies involving human participants were reviewed and approved by the Kardan Research Ethics Committee. The ethics committee waived the requirement of written informed consent for participation.

## Author Contributions

MR took the overall responsibility of the manuscript and wrote the Introduction part. SJ identified the research gap and the technical help during the whole process. MA wrote the Literature Review and Discussion Part. HG completed the Methodology, Analysis, and Interpretation part. All authors contributed to the article and approved the submitted version.

## Conflict of Interest

The authors declare that the research was conducted in the absence of any commercial or financial relationships that could be construed as a potential conflict of interest.

## Publisher’s Note

All claims expressed in this article are solely those of the authors and do not necessarily represent those of their affiliated organizations, or those of the publisher, the editors and the reviewers. Any product that may be evaluated in this article, or claim that may be made by its manufacturer, is not guaranteed or endorsed by the publisher.
